# Cultured Human Fibroblast Biostimulation Using a 940 nm Diode Laser

**DOI:** 10.3390/ma10070793

**Published:** 2017-07-13

**Authors:** Rebeca Illescas-Montes, Lucía Melguizo-Rodríguez, Francisco Javier Manzano-Moreno, Olga García-Martínez, Concepción Ruiz, Javier Ramos-Torrecillas

**Affiliations:** 1 Biomedical Group (Bio277), Department of Nursing, Faculty of Nursing, University of Granada Campus from Melilla, C/Santander, 1, Melilla 58071, Spain; rebecaim@ugr.es; 2 Instituto de Investigación Biosanitaria ibs.GRANADA, University of Granada, C/Doctor Azpitarte 4, planta, Granada 18012, Spain; luciamr@ugr.es (L.M.-R.); fjmanza@ugr.es (F.J.M.-M.); ogm@ugr.es (O.G.-M.); jrt@ugr.es (J.R.-T.); 3 Biomedical Group (Bio277), Department of Nursing, Faculty of Health Sciences, University of Granada, Avda de la Ilustración 60, Granada 18016, Spain; 4 Biomedical Group (Bio277), Department of Stomatology, School of Dentistry, University of Granada, Campus de Cartuja s/n, Granada 18071, Spain; 5 Institute of Neuroscience, University of Granada, Granada Health-Science Technology Park, Armilla, Granada 18100, Spain

**Keywords:** wound healing, fibroblasts, diode laser, biostimulation, cell culture

## Abstract

Background: Fibroblasts are the main cells involved in regeneration during wound healing. The objective was to determine the effect of 940 nm diode laser on cultured human fibroblasts using different irradiation regimens. Methods: The CCD-1064Sk human epithelial fibroblast cell line was treated with a 940 nm diode laser at different energy doses (power: 0.2–1 W and energy density: 1–7 J/cm^2^) using different transmission modes (continuous or pulsed). The effect on cell growth at 24 and 72 h post-treatment was examined by measuring the proliferative capacity, the impact on the cell cycle, and the effect on cell differentiation. Results: fibroblast proliferative capacity was increased at 24 and 72 h post-treatment as a function of the energy dose. The greatest increase was observed with a power of 0.2 or 0.5 W and energy density between 1 and 4 J/cm^2^; no difference was observed between continuous and pulsed modes. There were no significant differences in cell cycle between treated groups and controls. α-actin expression was increased by treatment, indicating enhanced cell differentiation. Conclusion: The 940 nm diode laser has biostimulating effects on fibroblasts, stimulating proliferative capacity and cell differentiation without altering the cell cycle. Further researches are necessary to explore its potential clinical usefulness in wound healing.

## 1. Introduction

Lasers are widely used for surgical/thermal (High Intensity Laser Therapy, HILT) or other therapeutic (Low Level Laser Therapy, LLLT) purposes [1,2]. The therapeutic usefulness of LLLT resides in the trophic, anti-inflammatory, and analgesic effects [3,4,5] that result from the transformation of light energy into biochemical energy [6,7]. Different types of Low Level Laser are used by their biostimulatory properties including solid lasers such as Nd:YAG, gas lasers as Helium Neon (HeNe) or CO_2_, liquid lasers, diode semiconductor lasers where the most common are gallium arsenide (GaAs), aluminum gallium arsenide (GaAlAs), indium gallium arsenide (InGaAs), and indium gallium arsenide phosphide (InGaAsP). These lasers operate in different modalities: continuous wave (CW) or pulsed (CP) and they are very effective in the application in soft tissue with excellent effect in wound healing [8]. Various studies have demonstrated that Low Level Laser Irradiation (LLLI) can increase cell proliferation, differentiation, and migration in tissues through its stimulation of growth factor and cytokine synthesis, thereby enhancing their regenerative capacity [9,10,11,12]. The therapeutic potential of the effects of LLLI on fibroblasts has been proposed, but there is a lack of conclusive supporting data or consensus on the most appropriate treatment protocol, which should consider the laser emission wavelength, energy power and density, and even the possibility of combining red spectrum and infrared wavelengths [13,14,15].

Fibroblasts are an essential cell component of soft tissue and are the main cells involved in wound healing, contributing to the restoration of tissue physiology by providing growth factors and extracellular matrix proteins [16]. Soft tissue lesions represent an important healthcare problem, especially chronic lesions, given their prevalence, the healthcare costs involved, and their repercussion on patient quality of life [17]. The search for novel approaches to the regeneration of damaged tissue is therefore of major clinical interest.

The growth and differentiation of osteoblasts, responsible for bone formation and regeneration, are known to be enhanced by 940 nm diode laser treatment, and this effect has been attributed to the release of autocrine factors (e.g., TGFβ1 or BMP-2) by osteoblasts in response to the irradiation [18,19,20]. However, no data are available on the effects of this treatment on other cell populations, such as fibroblasts.

Besides its potential therapeutic value and non-invasive application, a further advantage of the 940 nm diode laser is its small size and easy handling. Hence its utility in daily clinical intervention is viable.

The objective of this study was therefore to determine the effects of different cellular parameters (growth, cell cycle, and expression of surface markers) on cultured human fibroblasts treated with this laser using different irradiation regimens, combining various values of power, energy density, and modes of irradiation (continuous and pulsed).

## 2. Results

### 2.1. Effect of a 940 nm Diode Laser on Fibroblast Growth

**Figure 1:**
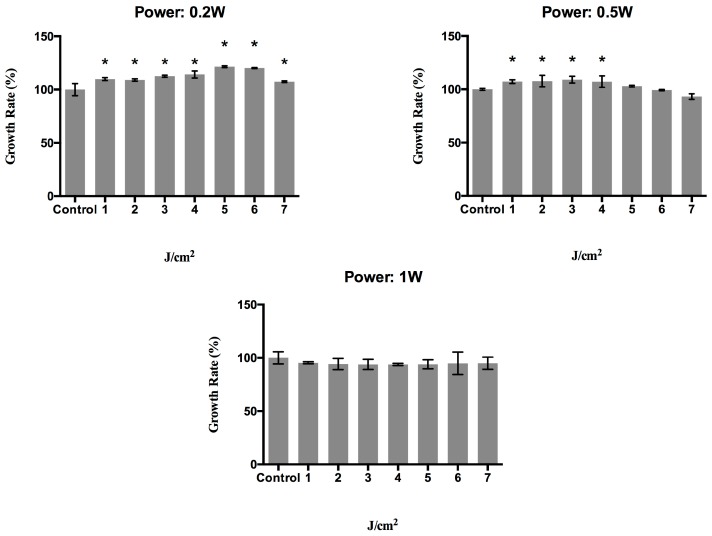
Growth rate (%) of human epithelial fibroblasts at 24 h post-irradiation with a 940 nm diode laser in continuous mode with different energy density (1, 2, 3, 4, 5, 6, and 7 J/cm^2^) and power (0.2, 0.5, and 1 watts) doses. * *p* < 0.05.

In an initial screening study, the optimal irradiation regimen was determined for fibroblast biostimulation with a 940 nm diode laser. The proliferative effect on the CCD-1064SK human epithelial fibroblast cell line was evaluated at 24 h after treatment in CW mode with different combinations of power values (0.2, 0.5, or 1 W) and energy density values (1, 2, 3, 4, 5, 6, or 7 J/cm^2^). Figure 1 depicts the results obtained, showing a statistically significant increase in proliferation for cells treated with a power of 0.2 W at the different energy densities with respect to non-treated cells (control group); this increase was dose-dependent, except at the highest energy density studied (7 J/cm^2^). When the power was 0.5 W, the proliferative capacity was significantly increased at densities of 1, 2, 3, and 4 J/cm^2^, but was reduced at densities above 4 J/cm^2^ until it reached similar values to those of the controls. Treatment with a power of 1 W decreased the growth rate at all energy densities studied.

**Figure 2:**
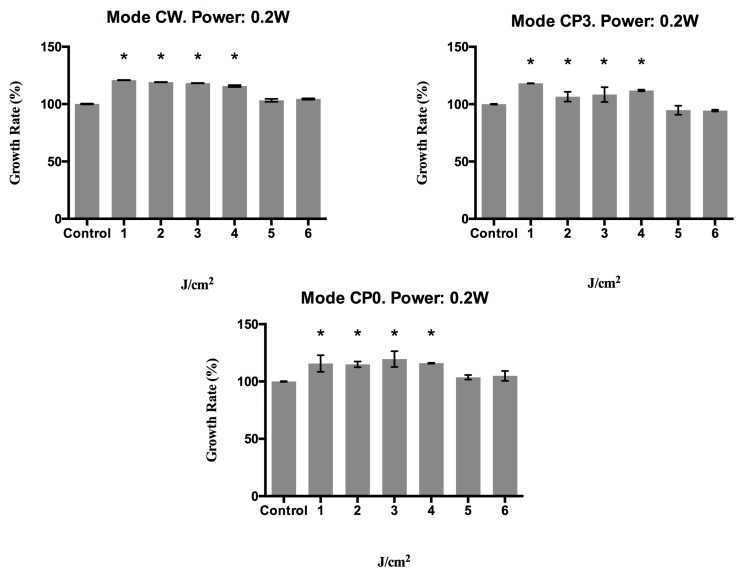
Growth rate (%) of human epithelial fibroblasts at 72 h post-irradiation with a 940 nm diode laser in different emission modes: CW, CP0, and CP3, with different energy density doses (1, 2, 3, 4, 5, and 6 J/cm^2^) and a power of 0.2 watts. * *p* < 0.05.

Based on the above findings, the proliferative capacity was determined at 72 h post-treatment with power values of 0.2 or 0.5 W and energy densities of 1, 2, 3, 4, 5, or 6 J/cm^2^, which were applied in continuous wave (CW) or pulsed mode with a duty cycle of 20% (CP0) or 50% (CP3). Figure 2 depicts the results obtained at 72 h after applying the different treatments, showing a significant (*p* < 0.003) growth increase versus controls in cells treated with a power of 0.2 W, regardless of the transmission mode (CW, CP0, or CP3), except at energy densities of 5 or 6 J/cm^2^, when there was no significant change in growth rate.

**Figure 3:**
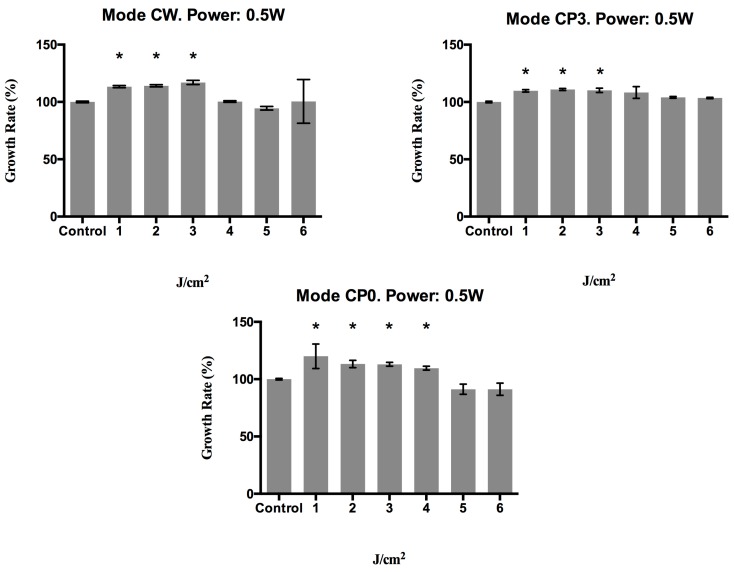
Growth rate (%) of human epithelial fibroblasts at 72 h post-irradiation with a 940 nm diode laser in different emission modes: CW, CP0, and CP3, with different energy density doses (1, 2, 3, 4, 5, and 6 J/cm^2^) and a power of 0.5 watts. * *p* < 0.05.

Figure 3 shows the growth of fibroblasts treated with a power of 0.5 W using the different energy transmission modes. In CW mode, the growth rate was significantly (*p* < 0.001) higher versus controls after treatment at 1, 2, or 3 J/cm^2^. In CP0 and CP3, the growth rate was also significantly (*p* < 0.032) higher at 4 J/cm^2^.

### 2.2. Effect of a 940 nm Diode Laser on Fibroblast Cell Cycle

**Figure 4:**
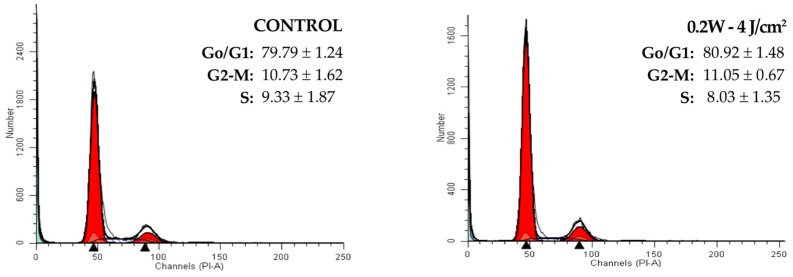

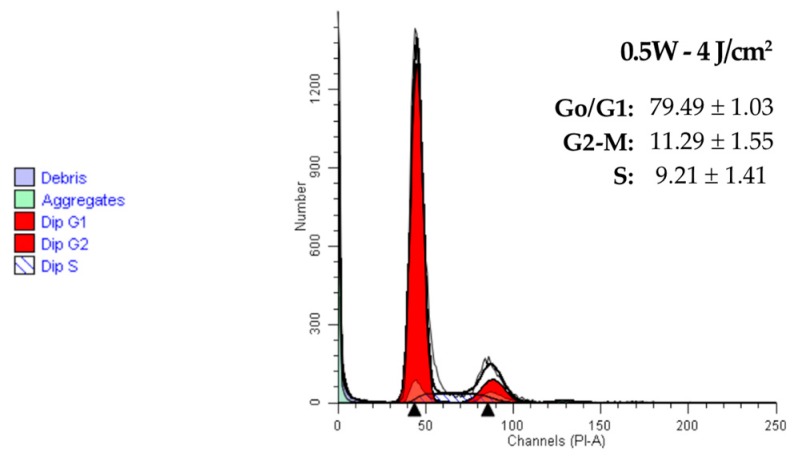
Fluorescence profile of cell cycle of human epithelial fibroblasts in culture at 24 h post-irradiation with a 940 nm diode laser. Controls were not treated. G0/G1, S, and G2/M represent the percentage of cells distributed among these phases after the treatment, as determined by flow cytometry.

Figure 4 exhibits cell cycle results for the CCD-1064Sk line at 24 h after treatment with a power of 0.2 or 0.5 W and energy density of 2, 3, or 4 J/cm^2^. No significant changes were detected between treated and non-treated cells in the percentage of cells in each cell cycle phase. In all cases, regardless of the energy applied, the cell cycle was normal and no DNA aneuploidy or signs of malignant transformation were observed.

### 2.3. Effect of a 940 nm Diode Laser on Fibronectin and α-Actin Expression in Fibroblasts

**Figure 5:**
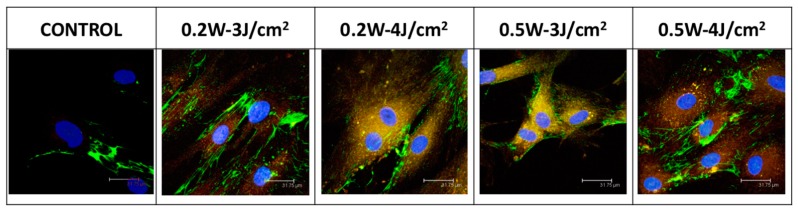
Immunostaining with fibronectin-fluorescein α-actin–phycoerythrin of human epithelial cells at 24 h post-irradiation with 940 nm diode laser under different conditions.

Immunofluorescence was used to determine the effect of the laser treatment on α-actin and fibronectin expression at 72 h. Expressions of α-actin and fibronectin were intense in cells treated in CW mode with powers of 0.2 or 0.5 W and energy densities of 3 or 4 J/cm^2^. Fibronectin but not α-actin was expressed by non-treated cells (Figure 5). Likewise in this figure we can observe that the treatment produces morphological changes typical of the differentiated cells, as a more rounded morphology.

## 3. Discussion

The biostimulatory effects of LLLI on cell populations are influenced by the type of laser, the emission wavelength, energy dose, and transmission mode, and it is necessary to establish the optimal treatment parameters for different lasers. The present study demonstrates that fibroblast growth can be stimulated by application of the 940 nm diode laser under specific treatment conditions (mode, power, and energy density). The benefits of this type of laser include its small size and easy handling.

Thus, fibroblast proliferative capacity was stimulated by treatment in CW mode with a power of 0.2 or 0.5 W, although this effect depended on the energy density applied. However, no biostimulatory effect on cell growth was observed after treatment with a power of 1 W. Hence, there is a power threshold above which the biostimulatory effect of irradiation disappears, as also reported by Huertas et al. [18] in their study of the effects of the same laser on the growth of MG63 osteoblast cells at 24 h. It can therefore be suggested that 940 nm diode laser irradiation has biostimulatory effects on human epithelial fibroblasts when the laser power is 0.2 or 0.5 W.

At 72 h of treatment, cell growth was lower at higher energy doses with both transmission modes, which may be attributable to fibroblast differentiation. This explanation is supported by the increased cell expression of α-actin, a classic marker of fibroblast differentiation into myofibroblasts, which are key cells in tissue healing [21]. In this regard, platelet-rich plasma (PRP) has been used in regenerative treatment and proposed as a useful option for wound-healing, especially in chronic cases [22,23,24]. PRP acts by supplying growth factors, notably TGF-β1, the main growth factor inducing the differentiation of fibroblasts into myofibroblasts. In vitro studies have reported a faster growth of fibroblasts after primary culture in the presence of PRP as the sole growth factor source than after culture in the presence of FBS. However, after a certain time period of culture with PRP, the cell growth stops and is even reversed in comparison to the same cells cultured with FBS. This reduced growth rate is connected to other changes in the morphological ultrastructure and marker expression of the cells, including an increase in α-actin expression. These cell changes indicate the differentiation of fibroblasts into myofibroblasts [25]. These data, together with the present findings, suggest that the action of the laser on fibroblasts may be similar to that of PRP, stimulating cell growth and inducing differentiation from fibroblasts into myofibroblasts above a certain dose or after a given time period, respectively. On the other hand, Ren et al. [26] suggested that the diode low-level laser showed positive effects on promoting fibroblast proliferation via changes in gene expression and the release of growth factors. Also, Hakki & Bozkurt [27] demonstrated that non-surgical laser applications modulate behavior of gingival fibroblasts inducing growth factors mRNA expressions and these applications can be used to improve periodontal wound healing.

Previous studies reported that growth of MG63 osteoblast-like cells (MG63) was induced by treatment with the same laser [18,20]. This effect appears to be related to increased gene expression of TGF-β1 and its receptors as a consequence of the laser treatment [19].

In a recent in vitro study, treatment of gingival fibroblasts with a 780 nm laser counteracted the adverse effects of proinflammatory cytokines, especially Interleukin 6 (IL-6), Interleukin 8 (IL-8), and Tumor Necrosis Factor α (TNFα), on this cell population in chronic wounds, such as the inhibition of growth, migration, or growth factor expression, thereby contributing to tissue regeneration [28]. The authors concluded that LLLI can stimulate cell function in oral mucosa wounds, even in the presence of high levels of inflammatory mediators. Within the limitations of in vitro studies, both their findings and the present results support the need for further research to explore the clinical potential of LLLI in tissue regeneration.

The results of the cell cycle study showed no significant differences between the laser treated-cells and controls in stage G0/G1, G2/M, or S. The cell cycle profile was normal in all cases, and no DNA aneuploid cells or signs of neoplastic transformation were found. This is a highly relevant finding, given the possible loss of control over growth and the malignization of cells that can result from the application of irradiation.

In conclusion, our data indicate that treatment with a 940 nm diode laser in either continuous or pulsed energy transmission mode stimulates fibroblast growth and induces differentiation in a simple and inexpensive manner, as long as the power of the laser does not exceed 0.5 W and the energy density is maintained between 1 and 4 J/cm^2^. Further research is necessary to define a therapeutic protocol, as suggested by other authors [15] and to elucidate the mechanisms underlying the biostimulating action of a 940 nm diode laser irradiation on fibroblasts and to investigate possible clinical applications.

## 4. Methods

### 4.1. Cell Culture

The human CCD-1064Sk epithelial fibroblast cell line was purchased from American Type Cultures Collection (ATCC, Manassas, VA, USA) (ATCC: CRL-2076) and maintained in Dulbecco’s Modified Eagle Medium (DMEM; Invitrogen Gibco Cell Culture Products, Carlsbad, CA, USA) with 100 IU/mL penicillin (Lab Roger SA, Barcelona, Spain), 50 μg/mL gentamicin (Braum Medical SA, Jaen, Spain), 2.5 μg/mL amphotericin B (Sigma, St. Louis, MO, USA), 1% glutamine (Sigma), and 2% HEPES (Sigma) supplemented with 10% fetal bovine serum (FBS) (Gibco, Paisley, UK). Cultures were kept at 37 °C in humidified atmosphere of 95% air and 5% CO_2_. Cells were detached from the culture flask with a solution of 0.05% trypsin (Sigma) and 0.02% ethylene diamine tetra-acetic acid (EDTA) (Sigma) and were then washed and suspended in complete culture medium with 10% FBS.

### 4.2. Laser Irradiation

This study used a diode laser (Biolase Technology, Inc., Irvine, CA, USA) that operates in the near infrared spectrum at a wavelength of 940 nm with maximum power output of 10 W and spot diameter of 400 microns. This device has a biostimulation hand piece that allows the light beam to be configured so that it touches the surface to be irradiated. This piece was used in all tests performed.

Cells were seeded in 24-well plates at an adequate between-well distance to avoid overlapping or scattered irradiation. After 24 h, cultures were pulse-irradiated at different power (0.2, 0.5, or 1 W) and energy density (1, 2, 3, 4, 5, 6, or 7 J/cm^2^) values, with the probe tip held at 1 cm from the cell layer. Three different treatment modes were used in each case: continuous wave (CW) or pulsed mode with a duty cycle of 20% (CP0) or 50% (CP3). The time of irradiation is determined by the energy density and power applied (in CW mode, for an energy density “a” it is required “x” seconds; in CP0 mode, for the same energy density it is required “4x” seconds; and for CP3 mode, it is required “2x” seconds). In all assays, the plate was uncovered during the irradiation, which was conducted at room temperature. Control cells underwent an identical procedure with the laser turned off.

### 4.3. Cell Proliferation Assay

After laser treatment under the above study conditions, cells were incubated for 24 or 72 h and cell proliferation was measured by MTT assay. Briefly, media were replaced with DMEM, without phenol-red, containing 0.5 mg/mL MTT (Sigma) and incubated for 4 h. Cellular reduction of the MTT tetrazolium ring resulted in the formation of a dark-purple water-insoluble deposit of formazan crystals. After incubation, the medium was aspirated and dimethyl-sulfoxide (DMSO) was added to dissolve the formazan crystals. Absorbance was then measured at 570 nm with a spectrophotometer (Sunrise™, Tecan, Männedorf, Switzerland). Results were expressed as % proliferation with respect to the control group, using the following formula:

% proliferation = (treated group OD570/control group OD570) × 100.

At least three experiments were conducted for each treatment, using the mean value in analyses.

### 4.4. Cell Cycle Assay

The cell cycle study of CCD-1064Sk fibroblast cell line was carried out by flow cytometry according to the procedure described by Manzano-Moreno et al. [29]. Before the treatment, we performed the starvation on cells for synchronization, for which cells were grown in medium DMEM without FBS. Briefly, fibroblasts were treated with a 940 nm diode laser in CW mode under different conditions (power of 0.2 or 0.5 W and energy density of 2, 3, or 4 J/cm^2^) for 24 h at 37 °C. Cells were then detached from the culture flask with a solution of 0.05% trypsin and 0.02% (w/v) (EDTA) and were washed and suspended in phosphate-buffered saline (PBS) at 2 × 10^4^ cells/mL; 2 mL of ice-cold 70% ethanol and 30% distilled H_2_O were vigorously added to 200 µL of the cell suspension. Cells were left for at least 30 min in ice. At the end of this period, cells were harvested by centrifugation and resuspended in 800 µL PBS. Cells were examined microscopically and, when clumped, were passed through a 25-gauge syringe needle. Cells were then incubated at 37 °C for 30 min with 100 µL of RNase (1 mg/mL) and 100 µL propidium iodide (PI). Finally, samples were analyzed using an argon-ion laser tuned to 488 nm (Fasc Vantage Becton Dickinson, Palo Alto, CA, USA), measuring forward and orthogonal light scatter and red fluorescence and determining both the area and peak of the fluorescent signal when possible. Results were expressed as percentage of cells in each phase (G0/G1, S, and G2/M).

### 4.5. Immunofluorescence

Fibroblasts were treated with the 940 nm diode laser in CW mode under different conditions (power of 0.2 or 0.5 W and energy density of 3 or 4 J/cm^2^) for 72 h at 37 °C. Immunostaining was performed on cells fixed in ice-cold methanol-acetone (1:1) for 10 min and then washed with PBS. Cells were subsequently blocked with FBS (10% in PBS) for 30 min and incubated for 2 h with the assayed mAbs (fibronectin and α-actin) at 1:500 dilution (Table 1). Nuclear counterstaining with 4,6-diamidino-2-phenylindole was performed after the removal of excess antibodies. Immunostaining was visualized using a Leica Spectral confocal laser microscope (Leica Microsystems GmbH, Wetzlar, Germany).

### 4.6. Statistical Analysis

SPSS v 22.0 software (SPSS Inc., Chicago, IL, USA) was used for the statistical analysis. Differences between means were evaluated using analysis of variance (ANOVA) and the Bonferroni procedure. Data are reported as means ± standard deviation (SD). Differences between experimental groups were considered statistically significant at *p* < 0.05.

## Figures and Tables

**Table 1:** Monoclonal antibodies (mAbs) used to study antigenic phenotype on ccd-1064sk line of fibroblast cells, with their specificity, the fluorochrome used to label the antibody, and the supplier.

mAbs	Fluorochrome	Supplier
Anti-human fibronectin fluorescein	FITC	R&D Systems (Minneapolis, MN, USA)
Anti-human α-actin	PE	R&D Systems (Minneapolis, MN, USA)
